# Supplementary material for: Design and Validity of a Choice-Modeling Questionnaire to Analyze the Feasibility of Implementing Physical Activity on Prescription at Primary Health-Care Settings
Source: Int J Environ Res Public Health. 2020 Sep 11;17(18):6627. doi: 10.3390/ijerph17186627 (PMC7559833; doi:10.3390/ijerph17186627)
Supplement: Supplementary file 1 [file ijerph-17-06627-s001.pdf]

Questionnaire S1: GPs´ questionnaire.

Sección 1 de 5

CUESTIONARIO MEDICINA

El presente cuestionario de 8-10 minutos de duración, elaborado por el Grupo de investigación ImFINE de la Universidad Politécnica de Madrid (UPM) perteneciente a la red EXERNET y el centro nacional "Exercise is Medicine". Pretende conocer las posibilidades de introducir la promoción y prescripción de actividad física (AF)/ejercicio físico (EF) en Atención Primaria.
Los datos se tratarán de forma anónima. Le agradecemos su máxima colaboración y su sinceridad en las respuestas.

Sección 2 de 5

Datos personales y profesionales:

Descripción (opcional)

Fecha: *

Mes, día, año

Edad: *

- 20-30
- 31-40
- 41-50
- 51-60
- 61-70

Sexo: *

- Hombre
- Mujer

Dirección asistencial: *

- ◯ Norte
- ◯ Noroeste
- ◯ Sur
- ◯ Sur-Este
- ◯ Este
- ◯ Oeste
- ◯ Centro

Grupo /Personal trabajador: *

	Estatutario fijo	Estatutario temporal l...	Estatutario temporal ...	Estatutario temporal ...
A1	◯	◯	◯	◯

Año de finalización de su carrera Universitaria: *

Introduzca 4 dígitos numéricos

Texto de respuesta corta

Años de experiencia profesional: *

Número entero de años (1 ó 2 dígitos, según proceda).

Texto de respuesta corta

¿Ha trabajado siempre en Atención Primaria? *

- ◯ Sí
- ◯ No

En caso negativo, indique los años que lleva trabajando en Atención Primaria

Responda solamente si respondió NO en la pregunta anterior.

Texto de respuesta corta

Conocimientos generales sobre promoción y prescripción de Actividad Física:

Descripción (opcional)

Terminología:

- Promoción: Acción y efecto de promover (RAE).
- Prescribir: Recetar, ordenar un remedio o tratamiento.(RAE).
- Tratamiento: Conjunto de medios que se emplean para curar o aliviar una enfermedad (RAE).
- Actividad física (AF): Cualquier movimiento corporal producido por los músculos esqueléticos, que exige gasto de energía (OMS).
- Ejercicio Físico (EF): Variedad de actividad física, estructurada, planificada y repetida, que se realiza en busca de un objetivo más específico relacionado con la mejora o el mantenimiento de uno o más componentes de la condición física y motriz.

1. La AF/EF posee efectos preventivos sobre la salud *

- ○ Si
- ○ No
- ○ NS/NC

2. ¿Qué colectivo de edad puede beneficiarse en mayor medida de la AF/EF?:

Puede marcar varias opciones

	20-30 años	31-49 años	50-65 años	> 65 años
Hombre	☐	☐	☐	☐
Mujer	☐	☐	☐	☐

3. ¿Cuándo se considera que una persona adulta es activa?: *

Puede marcar varias opciones

- ☐ Si realiza, al menos, 150 minutos/semana AF de intensidad moderada y además ejercicios de fuerza y flexibilidad, ...
- ☐ Si realiza, al menos, 75 minutos/semana AF de intensidad vigorosos y además ejercicios de fuerza y flexibilidad, al...
- ☐ Sólo si se somete a estímulos físicos que aumenten la condición física
- ☐ NS/NC

4. En la promoción y prescripción de actividad y ejercicio físico: *

	Si	No	NS/NC
La natación es el mejor ejerc...	○	○	○
En personas con obesidad e...	○	○	○
La electroestimulación pued...	○	○	○
El trabajo de fuerza, mediant...	○	○	○
Trabajar el equilibrio en pers...	○	○	○

[1]. La natación es el mejor ejercicio físico para la prevención de osteoporosis.
[2]. En personas con obesidad es aconsejable un trabajo de fuerza muscular.
[3]. La electro-estimulación puede ser una alternativa para trabajar la capacidad neuromuscular de un paciente con movilidad reducida.
[4]. El trabajo de fuerza, mediante Pilates, CORE, suelo pélvico, ¿Podría ayudar a prevenir la incontinencia urinaria en determinadas pacientes?
[5]. Trabajar el equilibrio en personas de edad avanzada podría disminuir lesiones por caídas.

Sección 4 de 5

Estado de Comportamiento:

Descripción (opcional)

5. Señale la frase con la que más se identifique, relacionada con AF y EF: *

- ○ No promociona, ni prescribe, ni pretende hacerlo
- ○ Tiene intención de promocionar y prescribir en los próximos meses
- ○ Se forma e interesa para promocionar y prescribir
- ○ Lleva promocionando y prescribiendo durante menos de 6 meses
- ○ Mantiene su promoción y prescripción como rutina durante más de 6 meses

Sección 5 de 5

Medicina

Responda a las siguientes preguntas:

6. ¿Se considera físicamente activo? *

- ○ Si
- ○ No

7. Conoce y utiliza los efectos preventivos de la AF para mejorar la salud de sus pacientes

Solamente puede seleccionar una opción

	NO los utilizo	SI los utilizo
SI Conozco efectos preventivos de la AF	☐	☐
NO conozco efectos preventivos de la ...	☐	☐

8. Su concienciación sobre los beneficios del EF favorece su uso en consulta *

- ◯ Si
- ◯ No
- ◯ NS/NC

9. ¿Cree que posee suficiente conocimiento para promocionar AF? *

- ◯ Si
- ◯ No

10. ¿Cree que posee suficiente conocimiento para prescribir EF? *

- Prescribir: Recetar, ordenar un remedio o tratamiento.(RAE).
- Ejercicio Físico (EF): Variedad de actividad física, estructurada, planificada y repetida, que se realiza en busca de un objetivo más específico relacionado con la mejora o el mantenimiento de uno o más componentes de la condición física y motriz.

- ◯ Si
- ◯ No

11. ¿Su colectivo debe liderar la promoción de AF en Atención Primaria? *

- Promoción: Acción y efecto de promover (RAE).
- Actividad física (AF): Cualquier movimiento corporal producido por los músculos esqueléticos, que exige gasto de energía (OMS).

- ◯ Si
- ◯ No

12. Si su respuesta anterior fue NO ¿Quién debería liderar?

Responder sólo si su respuesta anterior fue NO.

- ○ Médicos de otras especialidades
- ○ Enfermeros
- ○ Psicólogos
- ○ Fisioterapeutas
- ○ Médicos deportivos
- ○ Licenciados AF/deporte
- ○ Nutricionistas
- ○ Ninguno
- ○ Otra...

13. ¿Considera necesario colaborar con otros profesionales? *

Puede marcar varias opciones

- ☐ Médicos de otras especialidades
- ☐ Enfermeros
- ☐ Psicólogos
- ☐ Fisioterapeutas
- ☐ Médicos deportivos
- ☐ Licenciados AF/deporte
- ☐ Nutricionistas
- ☐ Ninguno
- ☐ Otra...

14. ¿Su colectivo debe liderar la prescripción de EF en Atención Primaria? *

- Prescribir: Recetar, ordenar un remedio o tratamiento.(RAE).
- Ejercicio Físico (EF): Variedad de actividad física, estructurada, planificada y repetida, que se realiza en busca de un objetivo más específico relacionado con la mejora o el mantenimiento de uno o más componentes de la condición física y motriz.

- ○ Si
- ○ No

15. Si su respuesta anterior fue NO ¿Quién debería liderar?

Responder sólo si su respuesta anterior fue NO.

- ◯ Médicos de otras especialidades
- ◯ Enfermeros
- ◯ Psicólogos
- ◯ Fisioterapeutas
- ◯ Médicos deportivos
- ◯ Licenciados AF/deporte
- ◯ Nutricionistas
- ◯ Ninguno
- ◯ Otra...

16. ¿Considera necesario colaborar con otros profesionales? *

Puede marcar varias opciones

- ☐ Médicos de otras especialidades
- ☐ Enfermeros
- ☐ Psicólogos
- ☐ Fisioterapeutas
- ☐ Médicos deportivos
- ☐ Licenciados AF/deporte
- ☐ Nutricionistas
- ☐ Ninguno
- ☐ Otra...

17. ¿Considera necesario colaborar con otros recursos de la Comunidad para una correcta promoción/prescripción de AF/EF? *

- ◯ Si
- ◯ No

18. En caso afirmativo. ¿Cuáles?

Responder sólo si su respuesta anterior fue SI. Puede marcar varias opciones.

- [ ] Ayuntamientos
- [ ] Centros Deportivos Municipales
- [ ] Centros de Fisioterapia
- [ ] Centros Wellness
- [ ] Consejería Juventud y Deportes
- [ ] Gimnasios privados
- [ ] Centros de enseñanza
- [ ] Ninguno
- [ ] Otra...

19. ¿Se formaría para promocionar AF y/o prescribir EF? *

- ○ Si
- ○ No

20. Considera necesaria la formación en otras áreas para poder optimizar la promoción de AF y/o prescripción de EF? *

- ○ Si
- ○ No

21. En caso afirmativo. ¿Cuáles?

Puede marcar varias opciones. Responder sólo si su respuesta anterior fue SI.

- [ ] Entrevista motivacional
- [ ] Gestión de tiempo
- [ ] Liderazgo
- [ ] Otra...

22. ¿Cómo preferiría la formación, en caso de considerarla necesaria? *

Puede marcar varias opciones

- ◯ Presencial
- ◯ On-line
- ◯ Mixta (Presencial /on-line)
- ◯ Otra...

23. Ha realizado formación sobre promoción de AF *

- ◯ Si
- ◯ No

24. En caso afirmativo. ¿Cuántos cursos de formación?

Responder sólo si su respuesta anterior fue SI.

0	1	2	3	4	5	6	7	8	9	10	
◯	◯	◯	◯	◯	◯	◯	◯	◯	◯	◯	Con más de 10 cursos, seleccione la casilla 10

25. Ha realizado formación sobre prescripción de EF *

- ◯ Si
- ◯ No

26. En caso afirmativo. ¿Cuántos cursos de formación?

Responder sólo si su respuesta anterior fue SI.

0	1	2	3	4	5	6	7	8	9	10	
◯	◯	◯	◯	◯	◯	◯	◯	◯	◯	◯	Con más de 10 cursos, seleccione la casilla 10

27. Considera necesario adaptar la herramienta de diagnóstico actual: *

- ◯ Si
- ◯ No

28. En caso afirmativo. ¿Para qué?

Puede marcar varias opciones. Responder sólo si su respuesta anterior fue SI.

- ☐ Clasificar patrón sedentarismo
- ☐ Valorar el tipo AF/EF
- ☐ Volumen AF/EF en tiempo
- ☐ Intensidad AF/EF
- ☐ Clasificar patrón AF
- ☐ Evolución histórica gráfica
- ☐ Analizar estadísticas del paciente
- ☐ Otra...

29. Clasifique de 1 (nada importante) a 6 (muy importante), según el grado de importancia que considere, las siguientes posibles BARRERAS para PROMOCIONAR y/o PRESCRIBIR ejercicio físico:

Debe seleccionar la opción que mejor se ajuste a su opinión

ESPACIOS físicos en su centro: *

	1	2	3	4	5	6	
nada importante	◯	◯	◯	◯	◯	◯	muy importante

Motivación de los PROFESIONALES del centro: *

	1	2	3	4	5	6	
nada importante	◯	◯	◯	◯	◯	◯	muy importante

GESTIÓN DEL TIEMPO *

	1	2	3	4	5	6	
nada importante	○	○	○	○	○	○	muy importante

Recursos MATERIALES y ECONÓMICOS: *

	1	2	3	4	5	6	
nada importante	○	○	○	○	○	○	muy importante

RELACIÓN EXTERNA (entre administraciones) *

	1	2	3	4	5	6	
nada importante	○	○	○	○	○	○	muy importante

MOTIVACIÓN/CONCIENCIACIÓN del paciente *

	1	2	3	4	5	6	
nada importante	○	○	○	○	○	○	muy importante

30. De entre las siguientes posibles SOLUCIONES, señale las que considere necesarias abordar para PROMOCIONAR y PRESCRIBIR ejercicio físico: *

	Si	No	NS/NC
Incorporar una nueva herra...	○	○	○
Disponer de espacios adecu...	○	○	○
Trabajo multidisciplinar entr...	○	○	○
Mejorar la formación contin...	○	○	○
Publicitar y promocionar est...	○	○	○
Implantación progresiva de ...	○	○	○
Dotación de recursos materi...	○	○	○
Fomentar el liderazgo en pro...	○	○	○
Utilizar la consulta de acogi...	○	○	○
Promover actividad física fu...	○	○	○
Dedicar más tiempo en cons...	○	○	○

*

[1]. Incorporar una nueva herramienta para evaluar el patrón de AF/sedentarismo del paciente a su historia clínica.
[2]. Disponer de espacios adecuados para evaluar el nivel de condición física del paciente.
[3]. Trabajo multidisciplinar entre profesionales.
[4]. Mejorar la formación continua de los profesionales.
[5]. Publicitar y promocionar estrategias de sensibilización del paciente.
[6]. Implantación progresiva de un modelo de promoción y prescripción de ejercicio físico.
[7]. Dotación de recursos materiales (i.e. acelerómetros, Apps telefonía móvil, etc.) para hacer un seguimiento y aumentar la motivación del paciente.
[8]. Fomentar el liderazgo en promoción de actividad física en los centros.
[9]. Utilizar la consulta de acogida para valorar inicialmente el patrón de AF/sedentarismo y/o el nivel de condición físico del paciente.
[10]. Promover actividad física fuera del centro, supervisada por profesionales del centro. (i.e. Rutas de paseos activos).
[11]. Dedicar más tiempo en consulta para promocionar actividad física.
